# Geniposidic Acid Targeting FXR “S332 and H447” Mediated Conformational Change to Upregulate CYPs and miR‐19a‐3p to Ameliorate Drug‐Induced Liver Injury

**DOI:** 10.1002/advs.202409107

**Published:** 2025-02-25

**Authors:** Minqi Fan, Yuanhang Xu, Bingxin Wu, Jiachan Long, Caihong Liu, Zuhui Liang, Rong Zhang, Zhongqiu Liu, Caiyan Wang

**Affiliations:** ^1^ State Key Laboratory of Traditional Chinese Medicine Syndrome International Institute for Translational Chinese Medicine Guangzhou University of Chinese Medicine Guangzhou 510006 China; ^2^ Chinese Medicine Guangdong Laboratory Hengqin Guangdong China

**Keywords:** bile acid metabolism, cholesterol production, drug‐induced liver injury, Farnesoid X receptor (FXR), miR‐19a‐3p

## Abstract

Drug‐induced liver injury (DILI), caused by chemical drugs and traditional Chinese medicine, often leads to severe outcomes like liver failure due to a lack of early detection markers. Farnesoid X receptor (FXR), a key regulator of bile acid (BA) and cholesterol metabolism, is a potential therapeutic target. This study investigates the pathogenesis, markers, and treatment strategies for DILI, focusing on the hepatoprotective effects of geniposidic acid (GPA) from *Gardenia jasminoides J. Ellis*. Using cellular and animal models of acute and chronic DILI induced by acetaminophen and triptolide, we explored GPA's mechanisms in BA and cholesterol metabolism. Lipidomic and BA analyses revealed that GPA alleviates DILI by enhancing bile acid synthesis and transport via FXR activation. Experiments using AAV‐shFXR, *Fxr* ^−^^/^^−^ mice and molecular assays demonstrated that GPA targets Ser332 and His447 on FXR ligand‐binding domain, promoting FXR nuclear translocation and initiating cytochrome P450 proteins (CYPs) transcriptional activation for BA metabolism. Additionally, miRNA sequencing and RNA‐pulldown assays showed that GPA‐activated FXR upregulates miR‐19a‐3p, binding to LXR 3'UTR to inhibit cholesterol production. These findings reveal the GPA‐FXR “structure‐target” relationship, highlighting a dual mechanism in which GPA promotes CYPs‐mediated bile acid metabolism and miR‐19a‐3p‐mediated cholesterol synthesis inhibition, providing a basis for FXR‐targeted DILI therapies.

## Graphical abstract

Drug abuse exacerbates cholesterol and bile acids burden, causing liver injury. Geniposidic acid targets FXR Ser332 and His447, together with SRC2‐3 recruitment and activated FXR nuclear translocation. Geniposidic acid‐activated FXR can upregulate CYPs to accelerate bile acids clearance and activate miR‐19a‐3p to suppress LXR‐mediated cholesterol synthesis, which improving cholesterol and bile acids metabolism in drug‐induced liver injury.

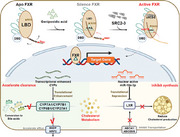

## 1 Introduction

The incidence of drug‐induced liver injury (DILI) has risen recently due to drug abuse, inappropriate clinical use, and lack of public awareness.^[^ ^1^ ^]^ The rapid and often concealed onset of DILI can lead to irreversible damage.^[^ ^2^, ^3^ ^]^ Significant causes of DILI include acetaminophen (APAP) abuse and long‐term use of the Chinese herbal medicine *Tripterygium wilfordii*.^[^ ^4^, ^5^, ^6^ ^]^ Both acute APAP‐induced and chronic *Tripterygium wilfordii*‐induced liver injuries are characterized by hepatocyte damage and imbalances in bile acid and lipid metabolism.^[^ ^7^ ^]^

High doses of drugs over short or long periods increase the metabolic burden on the liver, disrupting bile acid and cholesterol metabolism and transport. This results in excessive lipid deposition in hepatocytes, heightened inflammatory sensitivity, overactivation of inflammatory responses, and accelerated necrosis or apoptosis.^[^ ^8^ ^]^ Acute DILI shows significant elevations in ALT, AST, total bile acids, and cholesterol levels.^[^ ^9^, ^10^ ^]^ Chronic DILI, however, does not show significant increases in ALT and AST but maintains elevated intrahepatic bile acids and cholesterol levels.^[^ ^11^ ^]^ Current DILI treatment focuses on suppressing the inflammatory response through drug withdrawal and hepatoprotective drugs, enhancing the body's oxidative stress level.^[^ ^12^, ^13^ ^]^ However, no targeted drugs address the imbalance of bile acid and cholesterol metabolism in DILI. Targeting these metabolic pathways to improve the inflammatory response is crucial for understanding DILI's pathogenesis, development, and prognosis.

Farnesoid X receptor (FXR) is predominantly expressed in the liver and intestine and significantly regulates bile acid, cholesterol, glucose, and other metabolic pathways.^[^ ^14^ ^]^ Altered FXR function is linked to liver disease.^[^ ^15^ ^]^ In cholestatic liver disease, elevated secondary bile acids inhibit FXR, suppressing the bile salt export pump (BSEP) gene and exacerbating bile stagnation.^[^ ^16^ ^]^ In hypercholesterolemia, FXR upregulates peroxisome proliferator‐activated receptor alpha (PPARα), promoting fatty acid oxidation, reducing triglyceride synthesis, and inhibiting sterol regulatory element‐binding protein 1 (SREBP‐1) through the small heterodimer partner (SHP)‐mediated pathway, further inhibiting triglyceride and cholesterol synthesis.^[^ ^17^ ^]^ FXR, a ligand‐dependent transcription factor, consists of a DNA binding domain (DBD), hinge region, and ligand binding domain (LBD).^[^ ^18^ ^]^ Ligand binding induces a conformational change, recruiting steroid receptor coactivators (SRCs) to initiate transcription of target genes like CYP7A1, CYP27A1, CYP8B1, CYP7B1, and BSEP.^[^ ^19^ ^]^ Nuclear translocation of FXR is crucial for regulating cytochrome P450 proteins (CYPs) involved in bile acid and cholesterol metabolism.^[^ ^20^ ^]^

MicroRNAs (miRNAs) also regulate bile acid and cholesterol homeostasis, with FXR implicated in miRNA transcription.^[^ ^21^ ^]^ In non‐alcoholic fatty liver disease, FXR activation inhibits miR‐34a, reducing liver lipid deposition and restoring liver function.^[^ ^22^, ^23^ ^]^ Conversely, in obese mice, FXR activation increases miR‐802 expression, thereby inhibiting insulin resistance.^[^ ^24^ ^]^ The “FXR‐miRNA” pathway presents a novel approach to improving cholesterol homeostasis.^[^ ^25^, ^26^ ^]^

Geniposidic acid (GPA), an iridoid glycoside, is a major active constituent of the traditional Chinese medicine *Gardenia jasminoides J. Ellis*.^[^ ^27^, ^28^ ^]^ Studies have shown that GPA alleviates bile duct ligation‐induced cholestatic injury in mice by improving ALT, AST, and SOD levels.^[^ ^29^ ^]^ GPA can activate FXR to protect against α‐naphthyl isothiocyanate‐induced cholestatic liver injury, suggesting GPA's hepatoprotective role through enhanced bile acid and cholesterol metabolism.^[^ ^30^ ^]^ However, the precise targets and molecular mechanisms by which GPA regulates bile acid and cholesterol metabolism in DILI remain unclear.

This study hypothesizes that GPA targets FXR and activates its nuclear translocation to regulate bile acid and cholesterol metabolism, thereby ameliorating DILI. We established three liver injury models: acute APAP, acute TP, and chronic TP, to investigate the therapeutic effect of GPA on DILI and confirm the regulatory role of FXR in DILI. GPA activates FXR, upregulating CYP metabolic enzymes to promote cholesterol metabolism and miR‐19a‐3p to inhibit LXR‐mediated cholesterol production. Through these pathways, GPA synergistically reduces bile acid and cholesterol levels, improving the inflammatory response in DILI.

## 2 Result

### 2.1 Geniposidic acid (GPA) Ameliorated Inflammation in Two Acute Drug‐induced Liver Injury (DILI) Mouse Models

**Figure 1:**
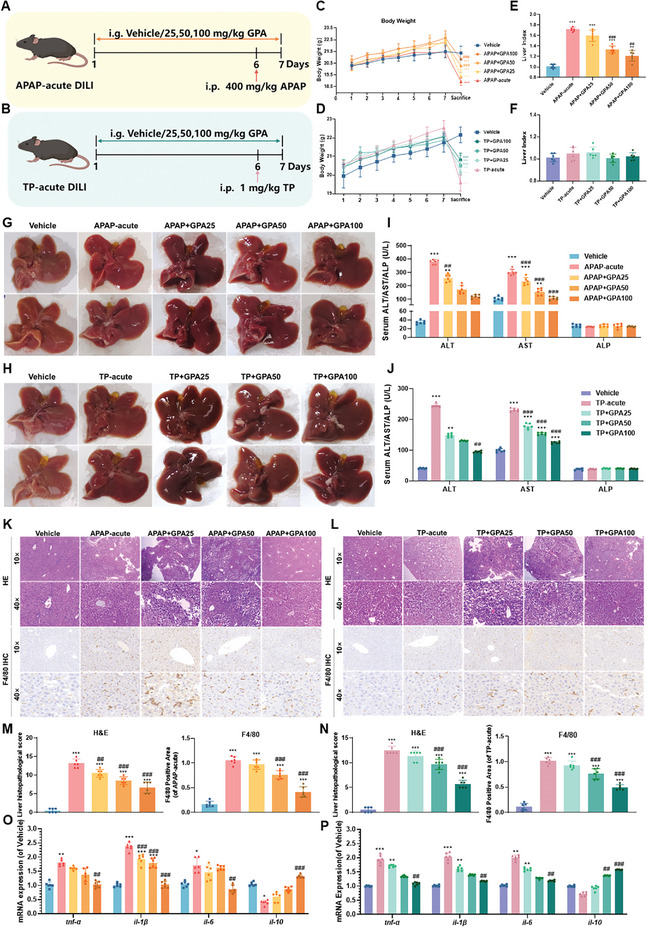
Evaluation of the efficacy of GPA in two mouse models of acute pharmacogenetic liver injury. A,B) Schematic of the mouse model of GPA‐treated APAP and TP acute DILI mice. C,D) Changes in body weight of mice in GPA‐treated APAP and TP acute DILI mice (*n *= 6). E,F) Liver index of GPA‐treated APAP and TP acute DILI mice (*n *= 6). G,H) Liver appearance of GPA‐treated APAP and TP acute DILI mice. I,J) Serum ALT, AST, and ALP in GPA‐treated APAP and TP acute DILI mice (*n *= 6). K,L) H&E staining and F4/80 immunohistochemical staining of the liver in mice with APAP and TP acute DILI treated with GPA. Scale bar: 50 µm. M,N) Ishak score and quantification of F4/80‐positive cells/total cells of liver from APAP acute DILI mice treated with GPA (*n *= 6). O,P) mRNA levels of hepatic inflammatory factors *tnf‐α*, *il‐1β*, *il‐6*, and *il‐10* in mice from GPA‐treated APAP and TP acute DILI mice (*n *= 6). All levels were measured by qPCR and normalized to GAPDH. All data are presented as means ± SD. Compared with control group, *P < 0.05, **P < 0.01, ***P < 0.001. Compared with model group, ^#^ *P < *0.05, ^##^ *P < *0.01, ^###^ *P < *0.001. All data are via one‐way analysis of variance (ANOVA).

Two mouse models of acute DILI were used to evaluate the potential therapeutic effect of GPA on DILI (**Figure** **1**A,B). GPA significantly protected against acute DILI‐induced weight loss in a concentration‐dependent manner (Figure 1C,D). In terms of liver index, APAP increased the liver weight ratio in mice compared to the vehicle group, and GPA administration reduced the APAP‐induced increase in liver weight ratio in a concentration‐dependent manner. However, no change was observed in the TP‐induced liver injury model (Figure 1E,F). The results of liver morphology observation showed that after GPA treatment, the liver became smooth and soft in texture, and the grey–white nodular particles were reduced (Figure 1G,H).

Serum levels of ALT and AST were significantly elevated after APAP or TP exposure, whereas ALP levels remained unchanged. Different doses of GPA reduced ALT and AST levels without affecting ALP levels (Figure 1I,J). H&E staining showed that more hepatocytes were damaged, GPA treatment reduced inflammatory infiltration and hepatocyte necrosis (Figure 1K–N). F4/80 positive staining, indicating Kupffer cell presence, was reduced by GPA, especially at 100 mg kg^−1^ (Figure 1K–N). RT‐qPCR analysis showed that GPA treatment decreased mRNA levels of pro‐inflammatory factors *tnf‐α*, *il‐1β*, and *il‐6*, and increased the anti‐inflammatory factor *il‐10*, compared to the vehicle group in both APAP and TP acute groups (Figure 1O,P). These results suggest that GPA ameliorates inflammatory injury caused by acute DILI.

### 2.2 GPA Improved Bile Acid and Cholesterol Metabolism Induced by Two Kinds of Acute DILI

**Figure 2:**
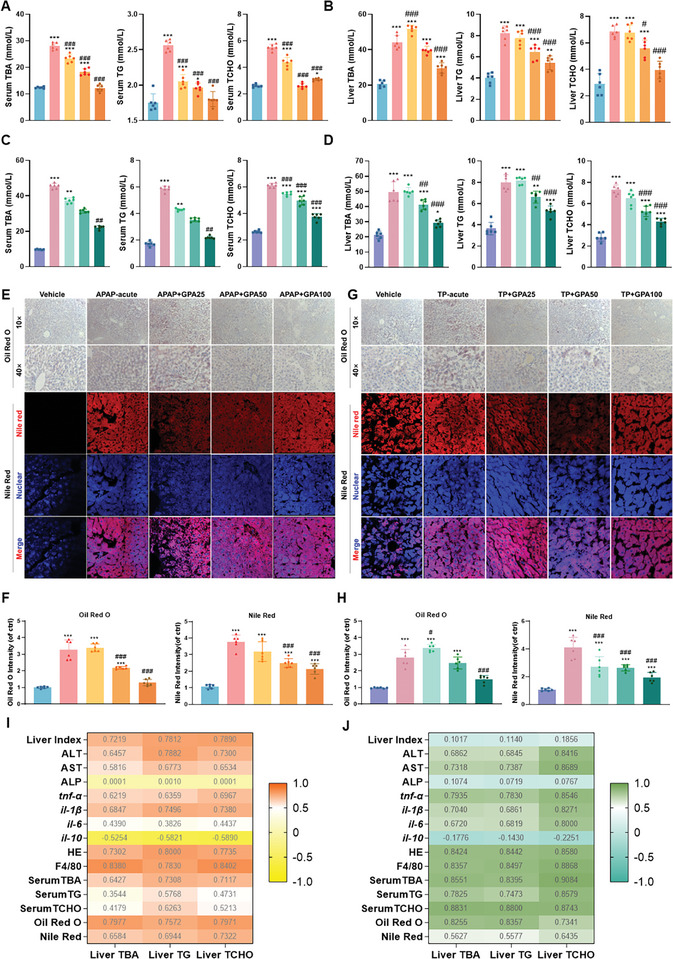
Evaluation of cholesterol and bile acid levels in two acute DILI mouse models by GPA. A,B) Serum and liver TBA, TG, and TCHO in GPA‐treated APAP acute DILI mice (*n *= 6). C,D) Serum and liver TBA, TG, and TCHO in GPA‐treated TP acute DILI mice (*n *= 6). E) Oil red O and Nile red staining of mouse liver from GPA‐treated APAP acute DILI mice. Scale bar: 50µm. F) Quantification of staining intensity in the liver from APAP acute DILI mice treated with GPA (*n *= 6). G) Oil red O and Nile red staining of mouse liver from TP acute DILI mice treated with GPA. Scale bar: 50 µm. H) Quantification of staining intensity in the liver from TP acute DILI mice treated with GPA (*n *= 6). I,J) Correlation analysis GPA‐treated acute APAP acute DILI and TP acute DILI mice (*n *= 6). All data are presented as means ± SD. Compared with control group, *P < 0.05, **P < 0.01, ***P < 0.001. Compared with model group, ^#^ *P < *0.05, ^##^ *P < *0.01, ^###^ *P < *0.001. All data are via one‐way analysis of variance (ANOVA).

The liver is crucial for bile acid and cholesterol metabolism, making it a key target for DILI treatment. Total bile acid (TBA), total triglyceride (TG), and total cholesterol (TCHO) levels are commonly used to assess liver metabolism. In the DILI models, serum levels of TBA, TG, and TCHO were elevated in the APAP‐acute and TP‐acute groups compared to the vehicle group (**Figure** **2**A,C). The increase in TBA and TG was more pronounced in the TP‐acute group, while TCHO levels were similarly elevated in both groups. GPA treatment significantly reduced serum TBA, TG, and TCHO levels in mice with acute DILI induced by APAP or TP (Figure 2A,C). Changes in liver TBA, TG, and TCHO levels mirrored those in serum (Figure 2B,D).

Biochemical indices confirmed that acute DILI caused bile acid and cholesterol metabolism disorders, and GPA treatment accelerated their metabolism, reducing serum and liver levels. Oil Red O staining showed fat vacuoles and lipid droplets in the livers of the APAP or TP acute model groups, which were reduced by GPA treatment (Figure 2E–H). Nile Red staining indicated significant liver lipid droplet deposition in the APAP or TP acute model groups, ameliorated by GPA intervention (Figure 2E–H). Pearson correlation analysis was performed to investigate the correlation between inflammation and cholesterol‐bile acid metabolism in DILI. In the APAP acute DILI model, liver bile acid and cholesterol levels positively correlated with liver inflammatory injury indicators and negatively correlated with the anti‐inflammatory factor *il‐10*, with no linear correlation to ALP (Figure 2I). Similar results were observed in the TP acute DILI model (Figure 2J). These findings indicate that GPA alleviates lipid deposition in acute DILI by enhancing lipid metabolism and reducing bile acid and cholesterol levels.

### 2.3 GPA Inhibits Inflammation, Bile Acid, and Cholesterol Levels in TP Mice with Chronic DILI

**Figure 3:**
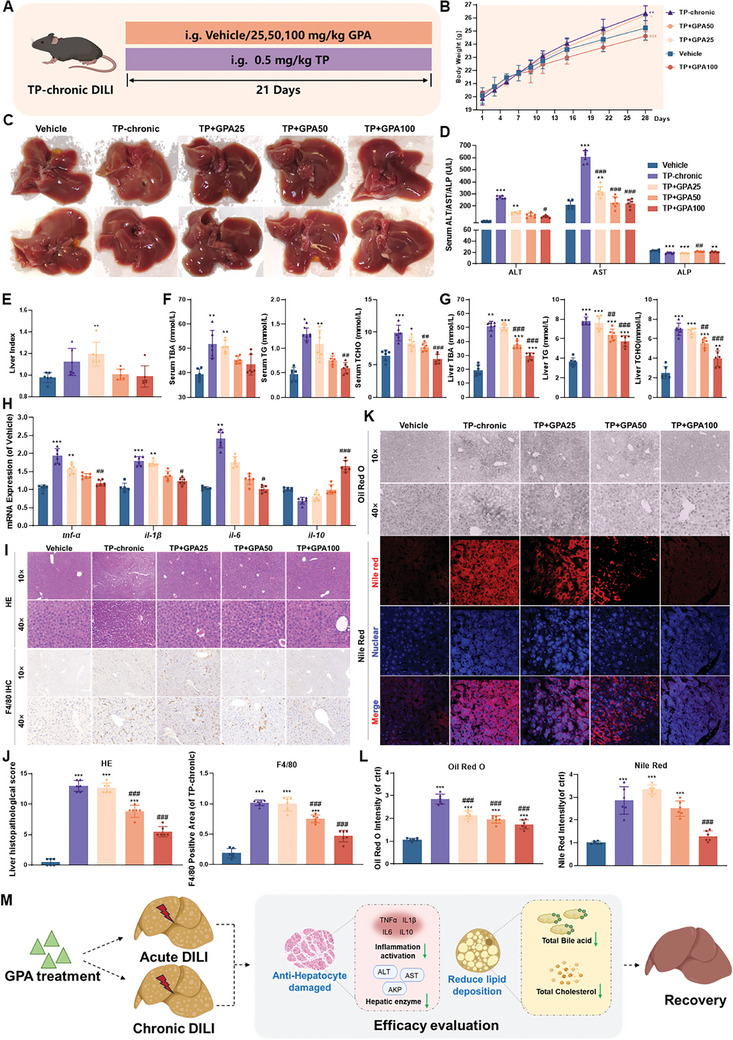
Evaluation of the efficacy of GPA in TP‐chronic DILI mouse. A) Schematic diagram of TP chronic DILI mouse model treated with GPA. B) Body weight changes in TP chronic DILI mice treated with GPA (*n *= 6). C) Appearance of the liver in TP chronic DILI mice treated with GPA. D) Serum ALT, AST, and AKP in GPA‐treated TP chronic DILI mice (*n *= 6). E) Liver index in GPA‐treated TP chronic DILI mice (*n *= 6). F,G) Serum and liver TBA, TG, and TCHO in TP chronic DILI mice treated with GPA (*n *= 6). H) Liver mRNA levels of inflammatory factors *tnf‐α*, *il‐1β*, *il‐6*, and *il‐10* in TP chronic DILI mice treated with GPA (*n *= 6). All levels were measured by qPCR, normalized to GAPDH. I) H&E staining and F4/80 immunohistochemical staining of the livers of TP chronic DILI mice treated with GPA. Scale bar: 50 µm. J) Ishak score and quantification of F4/80‐positive cells/total cells of liver from TP chronic DILI mice treated with GPA (*n *= 6). K) Oil red O and Nile red staining of mouse liver from TP acute DILI mice treated with GPA. Scale bar: 50 µm. L) Quantification of staining intensity in the liver from TP acute DILI mice treated with GPA (*n *= 6). M) Summary of the results of GPA treatment of acute and chronic DILI. Scale bar: 50 µm. All data are presented as means ± SD. Compared with control group, *P < 0.05, **P < 0.01, ***P < 0.001. Compared with model group, ^#^ *P < *0.05, ^##^ *P < *0.01, ^###^ *P < *0.001. All data are via one‐way analysis of variance (ANOVA).

Clinically, APAP‐induced DILI has an acute and rapid onset, rarely progressing to chronic disease. Therefore, we focused on TP‐mediated DILI in chronic liver disease, characterized by a long course and persistent disease. The specific protocol for TP‐mediated chronic DILI in mice is shown (**Figure** **3**A). Mice with TP‐induced chronic liver injury exhibited significantly increased body weight and liver index, which were significantly reduced by GPA treatment at various concentrations compared to the model group (Figure 3B,E), serum ALT and AST levels were significantly reversed by GPA treatment (Figure 3C). Liver biochemical parameters showed that GPA significantly reduced total bile acids, total cholesterol, and total triglycerides in TP‐induced chronic DILI mice (Figure 3F,G). GPA also reduced the mRNA expression of pro‐inflammatory cytokines *tnf‐α*, *il‐1β*, and *il‐6*, while promoting the expression of the anti‐inflammatory cytokine *il‐10* in mouse hepatocytes (Figure 3H). Additionally, GPA treatment reduced inflammatory infiltration (Figure 3I,J). Oil Red O and Nile Red staining of mouse liver tissue indicated that GPA significantly reduced lipid accumulation in TP chronic DILI cells (Figure 3K,L). These findings suggest that GPA ameliorates inflammation and hepatic bile acid and cholesterol accumulation in TP‐induced chronic DILI mice (Figure 3M).

### 2.4 GPA Promotes Cholesterol Metabolism and Clearance by Accelerating the Synthesis and Extrahepatic Transport of Primary Bile Acids

**Figure 4:**
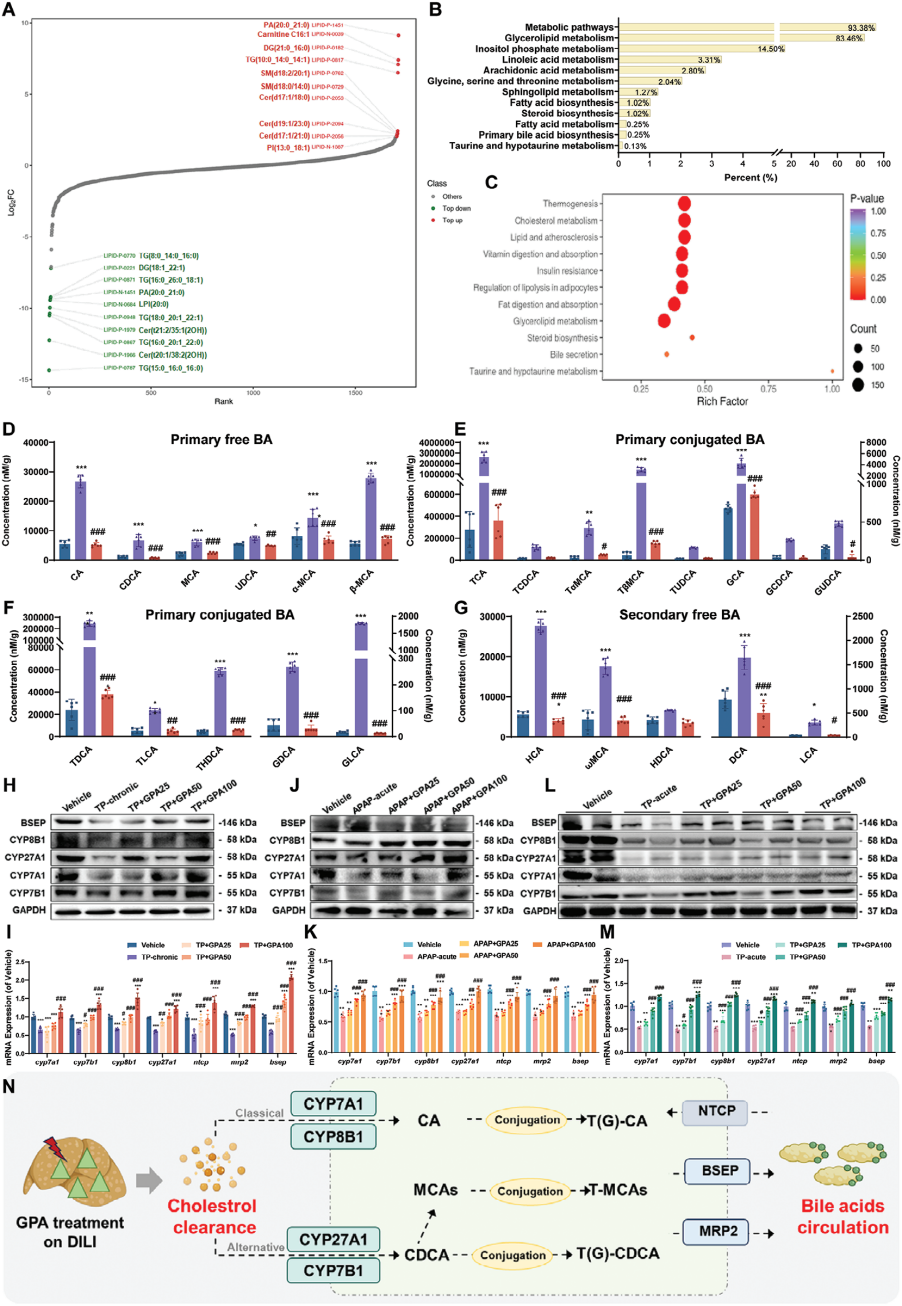
GPA promotes cholesterol metabolism by accelerating the synthesis and efflux of primary bile acids. A) Dynamic distribution map of differences in lipid content. B) Differential lipid KEGG classification map. C) Differential lipid KEGG enrichment map. D) Primary free bile acid content in mouse liver (*n *= 6). E) Mouse liver primary conjugated bile acid content (*n *= 6). F) Mouse liver secondary conjugated bile acid content (*n *= 6). G) Secondary free bile acid content in mouse liver (*n *= 6). H,I) The mRNA and protein expression of hepatic BSEP, CYP7A1, CYP8B1, CYP27A1, and CYP7B1 in TP chronic DILI mice treated with GPA (*n *= 6). All mRNA levels were measured by qPCR, normalized to GAPDH. J,K) The mRNA and protein expression of hepatic BSEP, CYP7A1, CYP8B1, CYP27A1, and CYP7B1 in APAP acute DILI mice treated with GPA (*n *= 6). All mRNA levels were measured by qPCR, normalized to GAPDH. L,M) The mRNA and protein expression of hepatic BSEP, CYP7A1, CYP8B1, CYP27A1, and CYP7B1 in TP chronic DILI mice treated with GPA (*n *= 6). All mRNA levels were measured by qPCR, normalized to GAPDH. N) Summary of upregulating CYPs‐transporter‐mediated primary bile acid synthesis and transport of GPA treatment of acute and chronic DILI. All data are presented as means ± SD. Compared with control group, *P < 0.05, **P < 0.01, ***P < 0.001. Compared with model group, ^#^ *P < *0.05, ^##^ *P < *0.01, ^###^ *P < *0.001. All data are via one‐way analysis of variance (ANOVA).

Both acute and chronic DILI are associated with elevated bile acid and cholesterol levels, while GPA counteracts DILI by reducing these levels in serum and liver. To elucidate the molecular mechanism of GPA's effect on DILI involving bile acid and cholesterol metabolism, we identified key metabolites using targeted lipidomics. It was found that GPA treatment could significantly reduce diglycerides (DGs) and triglycerides (TGs) (**Figure** **4**A). Enrichment KEGG analysis of differential metabolites in TP chronic liver injury highlighted enrichment in metabolic pathways and glycerolipid metabolism (Figure 4B). GO analysis indicated enrichment in cholesterol metabolism and bile secretion pathways, suggesting GPA may protect the liver by enhancing bile acid synthesis and excretion, thereby reducing serum cholesterol levels (Figure 4C). Together, these data suggested that glycerolipids may play an important role in the GPA treatment of DILI. Cholesterol is the smallest transport unit of glycerides the primary substrate for bile acid synthesis, and its hepatic clearance relies on bile acid metabolism. Targeted bile acid metabolomics revealed significantly increased levels of primary free bile acids (CDCA, CA, UDCA) and primary conjugated bile acids (TCDCA, GCDCA, TCA, GCA) in TP‐injured mice, which decreased after high‐concentration GPA treatment (Figure 4D,E). Secondary bile acids in TP‐injured mice were also reduced, with further decreases seen following GPA intervention (Figure 4F,G).

Given that chronic TP injury increases hepatic primary bile acid levels while GPA decreases both primary and secondary bile acid synthesis, it is likely that GPA facilitates hepatic cholesterol metabolism via bile acids, enhancing their excretion and reducing cholesterol and bile acid levels. We analyzed GPA's effect on primary bile acid synthesis pathways in TP chronic DILI mice. WB and qPCR results showed decreased protein and mRNA expression of CYP7A1, CYP7B1, CYP8B1, and CYP27A1 in TP chronic DILI mice, which GPA upregulated in a dose‐dependent manner, activating both classical and alternative bile acid synthesis pathways (Figure 4H,I). Similar upregulation of CYPs was observed in APAP acute DILI (Figure 4J,K) and TP acute DILI models (Figure 4L,M), indicating GPA's role in reducing cholesterol levels by promoting primary bile acid synthesis.

To determine whether GPA reduces bile acid levels by accelerating primary bile acid transport out of the liver, we examined the expression of transporter genes involved in bile acid excretion: BSEP and MRP2 (transporting conjugated primary bile acids out of the liver), and NTCP (transporting extrahepatic bile salts into hepatocytes) (Figure 4H–M). Both acute and chronic DILI models showed decreased expression of these transporters, which GPA intervention significantly increased, enhancing liver bile acid transport capacity (Figure 4N).

### 2.5 GPA Activates Farnesoid X Receptor (FXR) to Import into the Nucleus and Up‐Regulates the Expression of CYPs Key Enzymes to Promote the Synthesis of Primary Bile Acids

**Figure 5:**
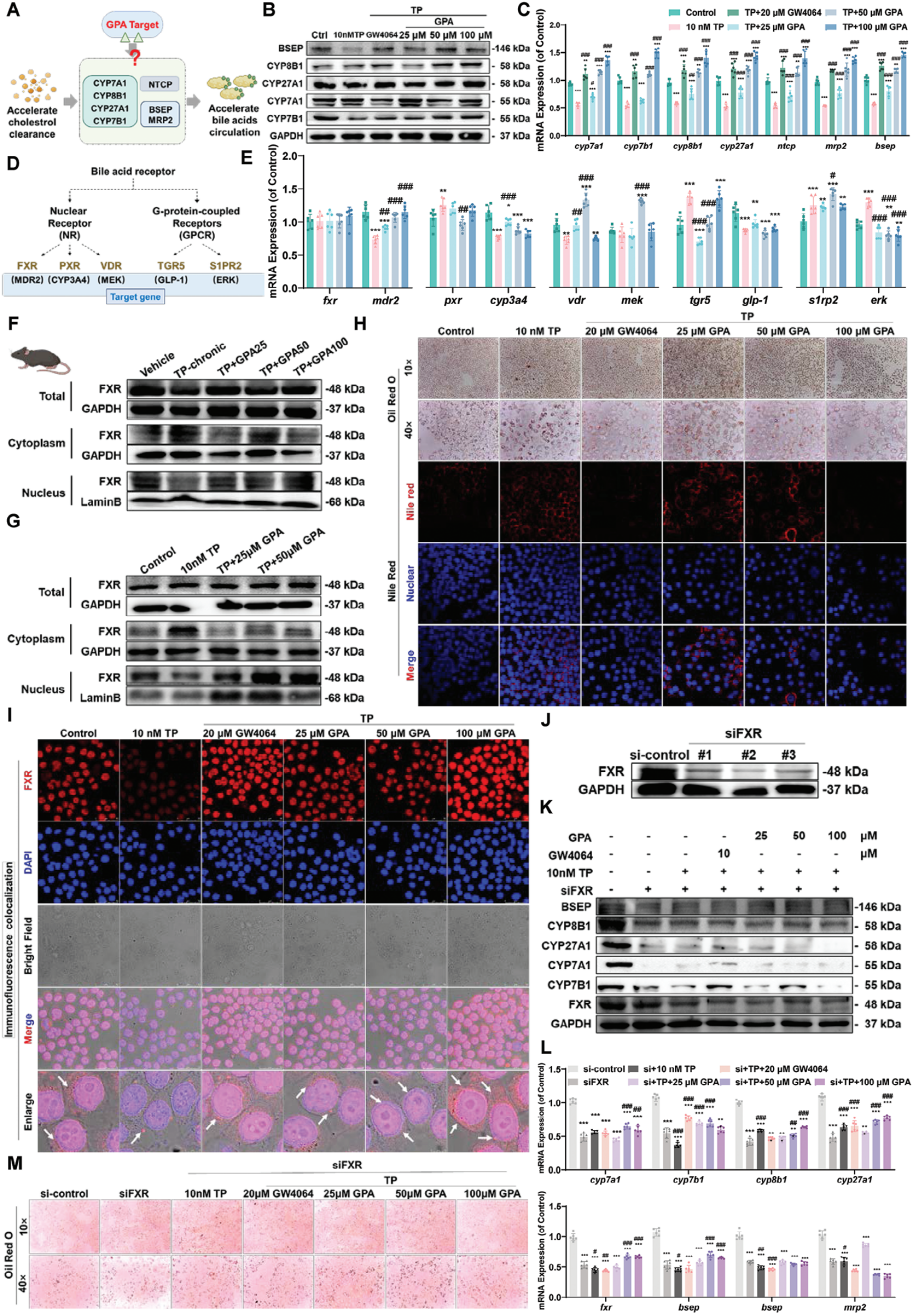
GPA activates FXR into the nucleus and up‐regulates CYPs key enzymes to promote bile acid synthesis and efflux. A) Hypotheses for upstream targets that regulate bile acid synthesis and transport. B,C) The mRNA and protein expression of BSEP, CYP7A1, CYP8B1, CYP27A1, and CYP7B1 in L02 hepatocytes induced by GPA treatment with TP (*n *= 6). All mRNA levels were measured by qPCR, normalized to GAPDH. D) Schematic representation of potential bile acid receptors that regulate bile acid synthesis and transport. E) mRNA expression of *fxr*, *tgr5*, *pxr*, *vdr*, *s1pr2*, and their target genes by in L02 hepatocytes induced by GPA treatment with TP (*n *= 6). All mRNA levels were measured by qPCR, normalized to GAPDH. F) Protein expression and distribution of hepatic FXR in TP chronic DILI mice treated with GPA. G) Protein expression and distribution of FXR in L02 hepatocytes induced by GPA treatment with TP. H) Oil red O staining with Nile red staining of L02 hepatocytes induced by GPA treatment with TP. I) Immunofluorescence co‐localization of TP‐induced L02 hepatocytes by GPA treatment. J) siFXR interferes with FXR expression in L02 cells. K,L) The mRNA and protein expression of BSEP, CYP7A1, CYP8B1, CYP27A1, and CYP7B1 in siFXR‐L02 hepatocytes induced by GPA treatment with TP (*n *= 6). All mRNA levels were measured by qPCR, normalized to GAPDH. M) Oil red O staining with Nile red staining of siFXR‐L02 hepatocytes induced by GPA treatment with TP. Scale bar: 50 µm. All data are presented as means ± SD. Compared with control group, *P < 0.05, **P < 0.01, ***P < 0.001. Compared with model group, ^#^ *P < *0.05, ^##^ *P <* 0.01, ^###^ *P < *0.001. All data are via one‐way analysis of variance (ANOVA).

To understand the mechanism of GPA in treating acute and chronic DILI, we used the TP‐induced L02 human hepatocyte injury model to explore GPA's therapeutic targets (**Figure** **5**A). GPA restored the protein and mRNA levels of CYP7A1, CYP7B1, CYP8B1, CYP27A1, and the transporters BSEP, MRP2, and NTCP, which were downregulated in TP‐induced liver cell injury (Figure 5B,C). These results suggest that GPA accelerates CYP and transporter‐mediated bile acid synthesis and transport in TP‐induced liver injury cells.

We examined upstream targets of GPA in regulating CYP enzymes and transporters, focusing on FXR, PXR, VDR, TGR5, and S1PR2^[^ ^31^ ^]^ (Figure 5D). RT‐qPCR results showed that GPA did not enhance FXR transcription but activated the expression of *mdr2*, an FXR target gene. There was no dose‐dependent transcriptional effect on *tgr5*, *pxr*, *vdr*, *s1pr2*, or their target genes, indicating GPA specifically activates fxr and initiates downstream transcription (Figure 5E).

In in vivo models of chronic DILI caused by TP, total cellular FXR protein expression remained unchanged, but GPA promoted FXR nuclear accumulation (Figure 5F). Similarly, we obtained the same results using in vitro models (Figure 5G). In the TP‐induced L02 hepatocyte injury model, GPA significantly reduced lipid droplets in a dose‐dependent manner, as shown by Oil Red O and Nile Red staining (Figure 5H). FXR immune fluorescence intensity and the organelles positioning reflect its therapeutic action. Immunofluorescence confirmed increased nuclear distribution of FXR with GPA treatment, consistent with WB results, indicating GPA promotes FXR nuclear translocation and activates gene expression of key CYP enzymes and transporters for bile acid synthesis (Figure 5I). To further investigate FXR's role, we used siRNA to inhibit FXR expression. We choose #2 as the key part of subsequent interference FXR expression sequences (Figure 5J). After the interference FXR expression, CYPs and BSEP protein expression decreased obviously (Figure 5K). CYPs and BSEP protein and gene transcription were further decreased by the addition of TP and interference with FXR expression, as confirmed by the increase in neutral lipid mass in oil Red O staining of the cells (Figure 5L,M). The ameliorative effect of GPA disappeared after FXR inhibition, confirming FXR as a key target for GPA (Figure 5J–M). These results suggest GPA activates FXR, upregulating key enzymes and transporters for bile acid synthesis, thereby promoting primary bile acid synthesis and alleviating DILI.

### 2.6 GPA Fails to Ameliorate TP‐Induced Hepatic Inflammation, Bile Acids, and Cholesterol Levels in FXR Knockout Mice

**Figure 6:**
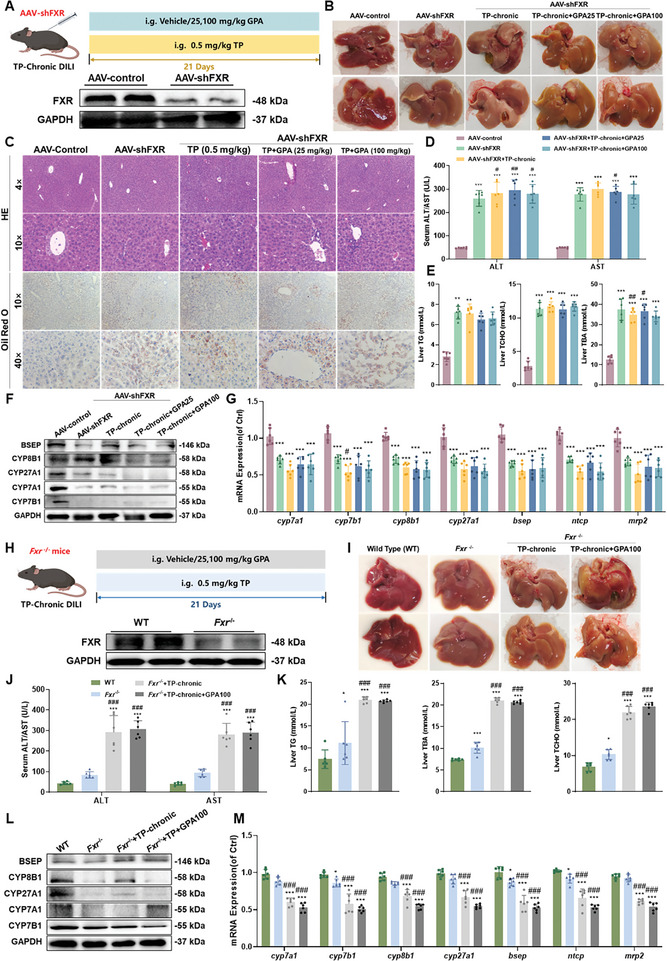
GPA fails to ameliorate TP‐induced hepatic inflammation, bile acids, and cholesterol levels in FXR knockout mice. A,B) Schematic and liver appearance of the TP chronic DILI AAV‐shFXR mouse treated with GPA. C) HE and Oil red O staining of the TP chronic DILI mouse model of AAV‐shFXR treated with GPA. Scale bar: 50 µm. D) Serum ALT and AST in GPA‐treated TP chronic DILI AAV‐shFXR mouse (*n *= 6). E) Liver TBA, TG, and TCHO in GPA‐treated TP chronic DILI AAV‐shFXR mouse (*n *= 6). F,G) The protein and mRNA expression of hepatic BSEP, CYP7A1, CYP8B1, CYP27A1, and CYP7B1 in GPA‐treated TP chronic DILI AAV‐shFXR mouse (*n *= 6). All mRNA levels were measured by qPCR, normalized to GAPDH. H,I) Schematic and liver appearance of the TP chronic DILI *Fxr^−/−^* mouse treated with GPA. J) Serum ALT and AST in GPA‐treated TP chronic DILI *Fxr^−/−^* mouse (*n *= 6). K) Liver TBA, TG and TCHO in GPA‐treated TP chronic DILI *Fxr^−/−^* mouse (*n *= 6). L,M) The protein and mRNA expression of hepatic BSEP, CYP7A1, CYP8B1, CYP27A1, and CYP7B1 in GPA‐treated TP chronic DILI *Fxr^−/−^* mouse (*n *= 6). All mRNA levels were measured by qPCR and normalized to GAPDH. All data are presented as means ± SD. Compared with control group, *P < 0.05, **P < 0.01, ***P < 0.001. Compared with model group, ^#^ *P < *0.05, ^##^ *P < *0.01, ^###^ *P < *0.001. All data are via one‐way analysis of variance (ANOVA).

In the DILI cell model, FXR was identified as the key target of GPA in treating DILI. To explore FXR's role in vivo, we used adenovirus‐mediated FXR gene silencing (AAV‐shFXR) and *Fxr^−/−^* mice (**Figure** **6**A,H). FXR protein expression in AAV‐shFXR mice liver was significantly inhibited compared to the control group (Figure 6A). In the AAV‐shFXR+TP chronic model group and those treated with GPA, the liver appeared reddish‐brown, slightly hard, and greasy on the cut surface, indicating attenuated therapeutic effects of GPA (Figure 6B).

H&E staining showed that GPA failed to reduce regional necrosis, inflammatory infiltration, and steatosis in AAV‐shFXR+TP chronic DILI mice, suggesting more severe liver injury and lipid deposition (Figure 6C). Consistent with oil red O staining results, GPA did not reverse inflammation, hepatocyte necrosis, and liver steatosis in these mice (Figure 6C). In the TP chronic DILI model, serum ALT, AST, liver TBA, TG, and TCHO were significantly increased in AAV‐shFXR+TP chronic model mice, with no significant difference compared to the AAV‐shFXR group, indicating aggravated liver injury (Figure 6D,E). GPA intervention failed to reduce these serum levels in AAV‐shFXR+TP chronic model mice, demonstrating GPA's lost therapeutic effect in the absence of FXR (Figure 6D,E).

The protective effect of GPA on TP chronic DILI in *Fxr* ^−/−^ mice was investigated using the same parameters as in WT mice (Figure 6H). GPA had no significant effect on liver injury in TP chronic DILI‐treated *Fxr* ^−/−^ mice, with abnormal bile acid and cholesterol metabolism (Figure 6I–K). WB and RT‐qPCR results showed that GPA intervention did not restore the inhibited protein and mRNA expression of CYP7A1, CYP7B1, CYP8B1, CYP27A1, BSEP, MRP2, and NTCP in AAV‐shFXR (Figure 6F,G). A similar situation was also observed in *Fxr* ^−/−^ mice (Figure 6L,M). These results suggest that GPA treats TP‐induced chronic liver injury by targeting FXR to regulate key hepatic CYP enzymes and transporters.

### 2.7 GPA Targeting of Serine 332 and Histidine 447 Residues of FXR Induces the Forming of the Active Conformation

**Figure 7:**
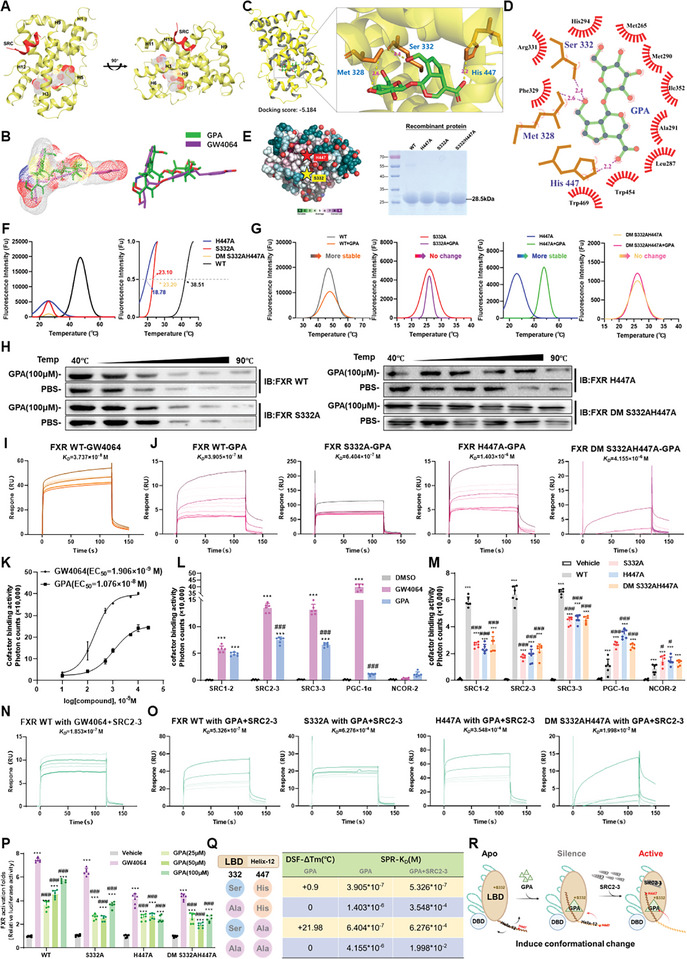
GPA targeting of S332 and H447 residues of FXR induces the forming of the active conformation. A) Schematic of the superposition of GPA and GW4064 bound to FXR LBD. B) Schematic of the superposition of GPA and GW4064 bound to the FXR LBD cavity. C) Demonstration of amino acid residues and hydrogen bonding distances for molecular docking of GPA with FXR. D) 2D lash diagram of GPA binding to FXR LBD. E) Conservation analysis of FXR and chromatogram of human FXR LBD wild‐type and mutant histones. F) Heat transfer curves of the wild type and mutant recombinant proteins of human‐derived FXR LBD. G) Heat transfer curves of GPA with human‐derived FXR LBD wild‐type versus mutant recombinant proteins. H) CETSA experiments of GPA with L02 cells overexpressing human FXR FL wild type versus mutant. I) SPR experiments of GPA, GW4064 with human‐derived FXR LBD wild‐type recombinant protein. J) SPR experiments of GPA with human‐derived FXR LBD mutant recombinant protein. K) Alphascreen experiments of GPA and GW4064 with human FXR LBD wild‐type recombinant protein. L,M) Alphascreen experiments of GPA with human FXR LBD wild type versus mutant recombinant protein screening for co‐binding factors (*n *= 6). N) SPR experiments of different doses of GW4064 with human‐derived FXR LBD wild‐type recombinant protein recruited to SRC2‐3. O) SPR experiments of different doses of GPA with human‐derived FXR LBD wild‐type and mutant recombinant proteins recruited for SRC2‐3. P) Dual‐luciferase reporter gene experiments of GPA with human‐derived FXR LBD wild type versus mutant recombinant protein (*n *= 6). *Bsep* activation levels were measured by luciferase reporter gene assay, normalized to vehicle group. Q) Summary of the combination of GPA and the wild type/mutant FXR. R) Assumption of GPA activates FXR conformation change. All data are presented as means ± SD. Compared with control group, *P < 0.05, **P < 0.01, ***P < 0.001. Compared with model group, ^#^ *P < *0.05, ^##^ *P < *0.01, ^###^ *P < *0.001. All data are via one‐way analysis of variance (ANOVA).

Using the crystal structure of the positive control GW4064 and FXR LBD (PDB: 3DCT) as a model, we performed molecular docking between GPA and FXR (**Figure** **7**A,B). The docking score of GPA with FXR LBD was ‐5.184 kcal mol^−1^, confirming GPA's targeting effect on FXR (Figure 7C). The binding structure of FXR LBD with GW4064 and GPA showed a three‐layer helical sandwich with GPA bound in the LBD pocket composed of helices H3, H5, H6, H7, H11, and H12 (Figure 7A). GPA binds to the same classical pocket region as GW4064, forming a T‐shaped hammer structure. However, the hammer handle binding cavity formed by GPA is shorter than that of GW4064 (Figure 7B). The hammerhead, formed by helices H4, H11, and H12, allows the hydroxyl group on GPA to form a hydrogen bond with the N–H moiety in His447 on helix H12, similar to the binding of GW4064 (Figure 7B,C).

Lash plot analysis revealed that GPA binds to the amino acid backbone of Met328 and to the side chains of Ser332 and His447. Due to GPA was combined with FXR Met328 main chain of carboxyl, which has nothing to do with the type of amino acid type. While GPA binds to Ser 332 and His447 side chains, we can adjust the size of GPA binding FXR cavity by changing the length of the side chains of Ser 332 and His447 (Figure 7D). We mutated amino acid residues around the FXR binding pocket based on the conservation of Ser 332 and His447 to assess their importance in GPA binding (Figure 7E). The S332A mutation increased the ligand binding pocket volume, preventing tight binding to FXR. The H447A mutation removed the imidazole group, crucial for hydrogen bonding. Recombinant proteins with human FXR LBD WT, S332A, H447A, and S332AH447A double mutations were expressed in vitro and showed no differences in molecular weight, suitable for subsequent experiments (Figure 7E).

Differential scanning fluorescence assay and mutant CETSA results showed that GPA improved the thermal stability of the recombinant FXR LBD protein (Figure 7F–H). DSF results showed that the stability of FXR LBD wild‐type protein was increased after adding GPA, and Ser332 mutation abolished the stability of GPA binding to FXR, suggesting that Ser332 is the key amino acid for GPA binding to FXR (Figure 7F,G). This result was also verified by the CETSA assay (Figure 7H). When Ser332 and His447 were co‐mutated, GPA's binding ability to FXR was reduced, and the thermal stability of FXR decreased. SPR showed that the KD value of GW4064 and FXR WT was 3.737 × 10^−8 ^M (Figure 7I), and a KD constant of 3.905 × 10^−7 ^M between GPA and FXR WT (Figure 7J), indicating that GPA and GW4064 have similar effects on combining FXR. Mutations in Ser332 and His447 reduced GPA's binding ability to FXR LBD, especially in the S332AH447A double mutant. In conclusion, GPA targets FXR LBD Ser332 and His447 residues to induce conformational changes, with stronger binding to His447 than Ser332. His447 may be the key residue for GPA to bind FXR and exert a conformational change.

AlphaScreen was used to screen the binding mode of GPA after forming an active conformation with FXR. The EC50 of GW4064 was 1.906 × 10^−9^ nm, while GPA bound FXR in a dose‐dependent manner, with an EC50 of 1.076 × 10^−8^ M (Figure 7K). GPA significantly induced the recruitment of FXR activators, such as SRC1‐2, SRC2‐3, and SRC3‐3, showing weaker PGC1α recruitment (Figure 7L). Although these recruitment abilities were weaker than those of the FXR‐GW4064 complex, the FXR‐GPA complex still formed an active conformation similar to the FXR‐GW4064 complex, recruiting coactivators. GPA also augmented the recruitment of the FXR corepressor NCoR‐2. AlphaScreen assays for GPA binding to cofactors in FXR LBD mutants demonstrated that, in the presence of SRC2‐3, the binding capacity of GPA to FXR LBD mutants was diminished compared to FXR LBD WT recombinant protein (Figure 7M). GPA's binding ability to FXR LBD S332A was weaker than to FXR LBD H447A, with the weakest binding ability in the S332AH447A double mutant (Figure 7N,O). The binding ability of the GPA‐FXR LBD complex to recruit coactivators decreased when Ser332 and His447 residues were mutated, with the greatest reduction observed in the S332A mutation. The significance of each residue was evaluated through a luciferase reporter assay of GPA agonism on FXR mutants, demonstrating that GPA significantly activated the transcription of FXR WT target gene promoters but showed weak transcriptional effects on S332A, H447A, and S332AH447A double mutant target gene promoters (Figure 7P). Summarizing the binding ability of GPA to FXR wild type and FXR mutant, we found that Ser332 of FXR was the key amino acid for GPA binding (Figure 7Q). Comprehensive GPA and FXR's ability to recruit co‐regulator results, it suggests that GPA can activate FXR via Ser332 and His447, initiating downstream transcription. Here, Ser332 is responsible for GPA binding, whereas His447 is responsible for closing the binding cavity of FXR (Figure 7R).

### 2.8 Activation of FXR by GPA Up‐Regulates miR19a‐3p Binding to LXR 3′UTR Involved in Cholesterol Synthesis

**Figure 8:**
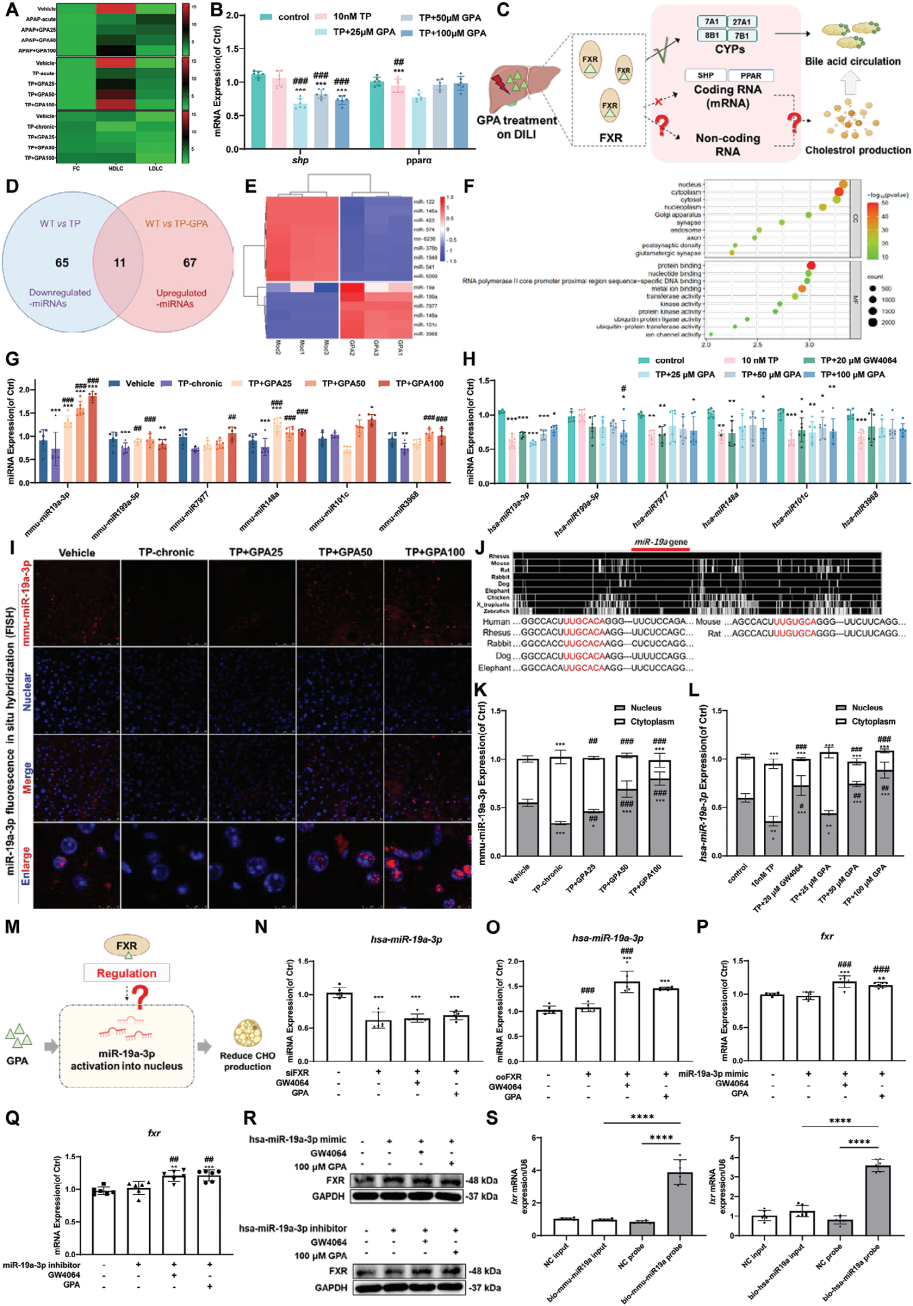
Activation of FXR by GPA upregulates miR19a‐3p binding to LXR 3′UTR involved in cholesterol synthesis. A) Serum FC, HDLC, and LDLC levels in APAP acute DILI mice, TP acute DILI mice, and TP chronic DILI mice (*n* = 6). B) Effect of GPA on hepatic *shp* and *ppar* mRNA levels in TP‐induced chronic DILI mice (*n *= 6). All mRNA levels were measured by qPCR, normalized to GAPDH. C) Schematic diagram of GPA‐targeted FXR regulation of non‐coding RNA‐mediated cholesterol metabolism. D) Wayne diagram of differentially expressed miRNAs. E) Differential miRNA target gene enrichment analysis. F) Differential miRNA target gene GO enrichment bubble diagram. G) miRNA expression in GPA‐treated TP chronic DILI mice (*n *= 6). miRNA levels were measured by qPCR, normalized to U6. H) miRNA expression in L02 cells from TP pharmacogenetic liver injury treated with GPA (*n *= 6). miRNA levels were measured by qPCR, normalized to U6. I) Hepatic miR‐19a‐3p probe FISH assay in GPA‐treated TP chronic DILI mice. Scale bar: 50 µm. J) Species conservation analysis of miR‐19a‐3p. K) Plasma nuclear expression of miR‐19a‐3p in GPA‐treated TP chronic DILI mice. Plasma miR‐19a‐3p levels were measured by qPCR, normalized to U6. Nuclear miR‐19a‐3p levels were measured by qPCR, normalized to Neat1. L) Plasma‐nuclear expression of miR‐19a‐3p in L02 cells from TP pharmacogenetic liver injury treated with GPA. Plasma miR‐19a‐3p levels were measured by qPCR, normalized to U6. Nuclear miR‐19a‐3p levels were measured by qPCR, normalized to Neat1. M) Assumption of the relationship between GPA activation of FXR and miR‐19a‐3p regulation. N) Expression of miR‐19a‐3p in siFXR‐L02 cells from TP drug‐derived liver injury treated with GPA (*n *= 6). miR‐19a‐3p levels were measured by qPCR, normalized to U6. O) Expression of miR‐19a‐3p in GPA‐treated TP drug‐derived liver injury oeFXR‐L02 cells (*n *= 6). miR‐19a‐3p levels were measured by qPCR, normalized to U6. P) mRNA expression of *fxr* in miR‐19a‐3p mimics‐L02 cells of TP DILI treated with GPA (*n *= 6). All mRNA levels were measured by qPCR, normalized to GAPDH. Q) mRNA expression of *fxr* in GPA‐treated TP pharmacogenetic liver injury miR‐19a‐3p inhibitor‐L02 cells (*n *= 6). All mRNA levels were measured by qPCR, normalized to GAPDH. R) Protein expression of FXR in GPA‐treated TP drug‐derived liver injury miR‐19a‐3p mimics‐L02 cells. S) Biotin‐labeled miR‐19a‐3p pull‐down assay in L02 and AML‐12 cells (*n *= 6). All mRNA levels were measured by qPCR, normalized to GAPDH. All data are presented as means ± SD. Compared with control group, *P < 0.05, **P < 0.01, ***P < 0.001. Compared with model group, ^#^ *P < *0.05, ^##^ *P < *0.01, ^###^ *P < *0.001. All data are via one‐way analysis of variance (ANOVA).

GPA treatment of acute and chronic DILI accelerates cholesterol clearance by activating FXR to enhance the metabolism and transport of primary bile acids. Thus, GPA may also directly inhibit cholesterol production by activating FXR. In three DILI mouse models, GPA maintained free cholesterol levels, increased liver HDLC, and decreased plasma LDLC in a dose‐dependent manner, suggesting that GPA protects against DILI by reducing intrahepatic cholesterol uptake and biosynthesis (**Figure** **8**A). We also found that GPA activation of FXR did not directly inhibit cholesterol production via the canonical SHP or PPARα pathways (Figure 8B). Therefore, we hypothesized that GPA activation of FXR might regulate cholesterol production genes by modulating non‐coding RNA, in addition to CYPs (Figure 8C).

miRNA sequencing of liver tissues from TP chronic DILI mice before and after GPA intervention identified 65 miRNAs downregulated in TP chronic DILI mice and 67 miRNAs upregulated in the GPA intervention group (Figure 8D). Eleven differentially expressed miRNAs were validated in cell and animal models (Figure 8E). Only hsa‐miR‐19a‐3p expression was decreased in the TP‐induced L02 hepatocyte injury model and significantly increased after GPA intervention in a dose‐dependent manner (Figure 8G,H). This suggests that miR‐19a‐3p plays a pivotal role in GPA‐mediated cholesterol regulation, improving both acute and chronic DILI.

The FISH assay demonstrated that GPA intervention facilitated the nuclear translocation of miR‐19a‐3p in a dose‐dependent manner (Figure 8I). Sequence alignment of miR‐19a‐3p from different species revealed high consistency among humans, primates, rabbits, dogs, and elephants, while mouse and rat sequences had two base differences from human miR‐19a‐3p (Figure 8J). Thus, both mouse and human cell models were used to validate the impact of miR‐19a‐3p on cholesterol production. GO functional enrichment analysis of differentially expressed miRNAs revealed involvement in nuclear and cytoplasmic components, protein binding, and nucleic acid binding (Figure 8F).

In TP chronic DILI mice and the TP‐induced L02 hepatocyte injury model, GPA intervention promoted miR‐19a‐3p nuclear localization in a concentration‐dependent manner (Figure 8K,L). We hypothesized that miR‐19a‐3p nuclear translocation is associated with FXR activation (Figure 8M). L02 cells transfected with FXR siRNA or overexpression plasmid showed that miR‐19a‐3p expression was inhibited after FXR inhibition, and neither pharmacological FXR agonist GW4064 nor GPA could restore miR‐19a‐3p expression (Figure 8N). After FXR overexpression, GPA upregulated miR‐19a‐3p expression, indicating that GPA activates miR‐19a‐3p after targeting FXR to form an active conformation (Figure 8O). FXR expression alone did not activate miR‐19a‐3p expression. miR‐19a‐3p mimics or inhibitors did not increase *fxr* mRNA expression (Figure 8P,Q). Similar results were obtained by Western Blot (Figure 8R). These results confirm that GPA targets FXR to form an active conformation, activating miR‐19a‐3p nuclear expression.

In a previous study, LXR was identified as a target gene of miR‐19a‐3p‐mediated cholesterol production, with *lxr* mRNA pulled down by miR‐19a‐3p, indicating that miR‐19a‐3p directly binds to *lxr* mRNA^[^ ^32^ ^]^ (Figure 8S). Our findings indicate that GPA targets FXR to upregulate miR‐19a‐3p expression, which binds to nuclear *lxr* mRNA to inhibit cholesterol production.

### 2.9 GPA Targets FXR to Activate Nuclear miR19a‐3p to Inhibit LXR‐Mediated Cholesterol Synthesis

**Figure 9:**
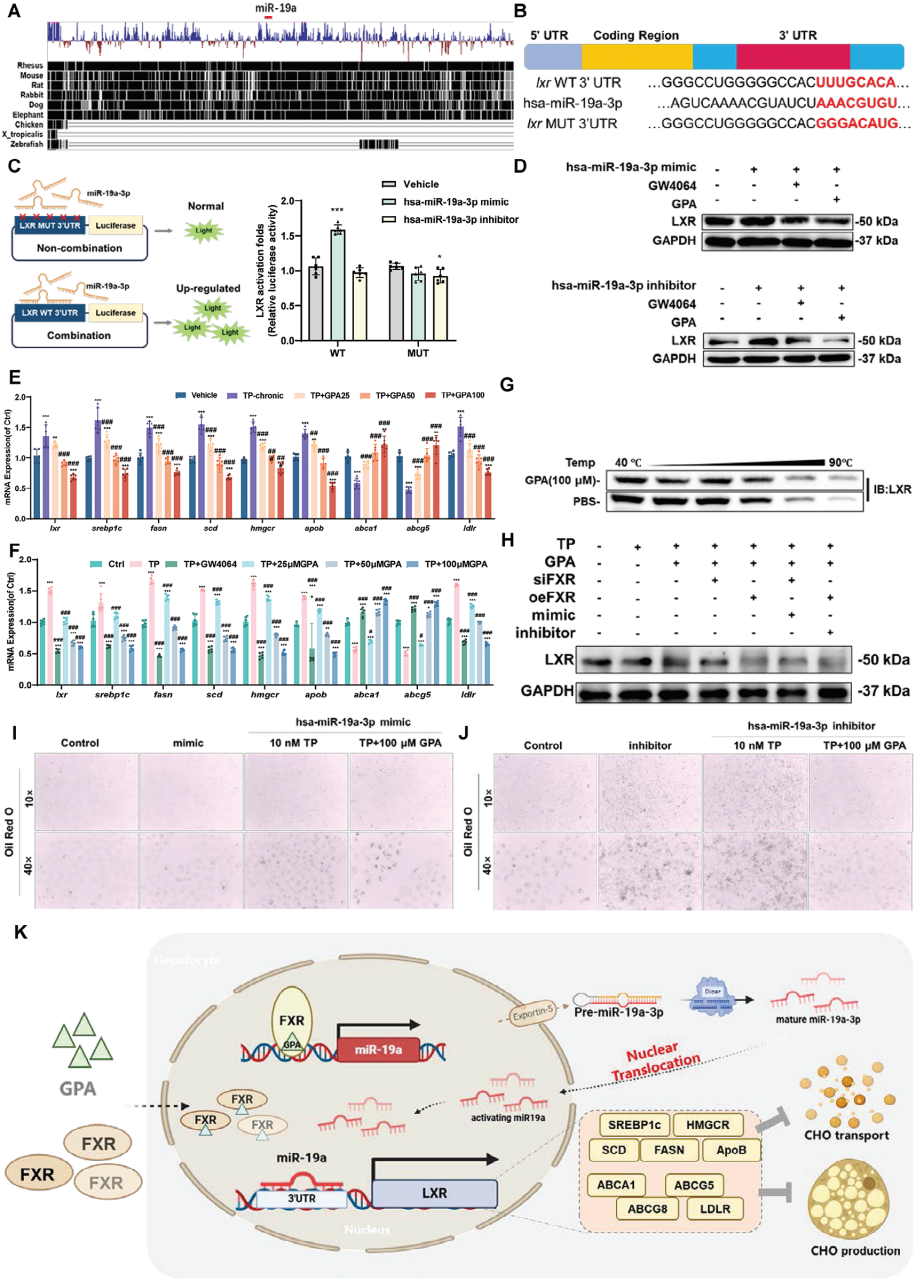
GPA targets FXR to activate nuclear miR19a‐3p to inhibit LXR‐mediated cholesterol synthesis. A) Conservation analysis of miR‐19a‐3p and LXR binding sites. B) Sequence of miR‐19a‐3p with LXR 3′UTR binding site in wild type and mutant. C) Luciferase reporter gene assay of miR‐19a‐3p with LXR 3′UTR binding wild type versus mutant (*n *= 6). *Lxr* activation levels were measured by luciferase reporter gene assay, normalized to vehicle group. D) Protein expression of LXR in L02 cells by miR‐19a‐3p mimics or inhibitors. E) mRNA expression of *lxr*, *srebp1c*, *fasn*, *scd*, srebp1c, fasn, scd, hmgcr, and apob in GPA‐treated TP chronic DILI mice (*n *= 6). All mRNA levels were measured by qPCR, normalized to GAPDH. F) mRNA expression of *lxr*, *srebp1c*, *fasn*, *scd*, srebp1c, fasn, scd, hmgcr, and apob in L02 cells of TP treated with GPA (*n *= 6). All mRNA levels were measured by qPCR, normalized to GAPDH. G) CETSA experiments of GPA with L02 cells overexpressing human LXR FL wild type. H) LXR protein expression of GPA or GW4064 with miR‐19a‐3p mimics/inhibitor‐L02 cells of TP DILI treated with GPA. I,J) miR‐19a‐3p mimics or inhibitor on Oil red O staining in L02 cells. K) Schematic representation of GPA targeting FXR to up‐regulate and activate miR‐19a‐3p into the nucleus to inhibit LXR activity and prevent cholesterol synthesis on DILI. Scale bar: 50 µm. All data are presented as means ± SD. Compared with control group, *P < 0.05, **P < 0.01, ***P < 0.001. Compared with model group, ^#^ *P < *0.05, ^##^ *P < *0.01, ^###^ *P < *0.001. All data are via one‐way analysis of variance (ANOVA).

A comparison of the binding regions of miR‐19a‐3p and *lxr* mRNA revealed that these regions are highly conserved among various genera (**Figure** **9**A). miR‐19a‐3p binds to the LXR 3′UTR region (Figure 9B). To confirm the binding site, we constructed WT and mutant binding site plasmids. Dual luciferase reporter gene experiments showed decreased binding ability of miR‐19a‐3p after mutation of the binding site, indicating an interaction between miR‐19a‐3p and the LXR 3′UTR region (Figure 9C).

WB results showed that overexpression of miR‐19a‐3p inhibited LXR protein expression, and the addition of GW4064 or GPA further inhibited FXR protein expression. Inhibition of miR‐19a‐3p promoted LXR protein expression, while GW4064 or GPA reduced LXR expression (Figure 9D). We also investigated the expression of LXR and downstream genes involved in cholesterol production and transport in animal and cellular models. Results showed that GPA inhibited the expression of LXR and lipid synthesis‐related genes *srebp1c*, *fasn*, *scd*, *hmgcr*, and *apob* in TP‐induced DILI animals and hepatocyte injury models. GPA also promoted the expression of cholesterol efflux transporter genes ABCA1 and ABCG5 and inhibited the expression of the cholesterol intrahepatic uptake gene LDLR, suggesting that GPA inhibits cholesterol synthesis and intrahepatic uptake in DILI while promoting extrahepatic cholesterol transfer (Figure 9E,F).

CETSA showed that GPA did not protect the thermal stability of LXR protein, indicating that GPA did not bind to LXR (Figure 9G). In the TP‐induced L02 cell model, LXR protein expression was decreased by the addition of GW4064 or GPA, and a miR‐19a‐3p inhibitor reversed the inhibitory effect on LXR (Figure 9H). This finding is consistent with cellular Oil Red O staining results, suggesting that GPA inhibits LXR‐mediated cholesterol production by upregulating miR‐19a‐3p expression after FXR activation, thereby reducing lipid accumulation in cells (Figure 9I,J). These results demonstrate that GPA targets FXR to activate miR‐19a‐3p in the nucleus, inhibiting LXR‐mediated cholesterol production and transport, further elucidating the mechanism by which GPA inhibits cholesterol production in DILI (Figure 9K).

## 3 Discussion

Bile acids are essential for fat metabolism and are predominantly found in the enterohepatic circulation, protecting through recycling, with a small proportion entering the peripheral circulation.^[^ ^31^ ^]^ They play a crucial role in cholesterol metabolism, with one‐third of cholesterol catabolism achieved through bile acid synthesis.^[^ ^33^ ^]^ Bile acids offer an important excretory pathway for cholesterol metabolism, with one‐third of cholesterol catabolism achieved through bile acid synthesis.^[^ ^34^ ^]^ Bile acids stimulate cholesterol secretion into bile, maintaining cholesterol solubility.^[^ ^35^ ^]^ Thus, bile acid‐cholesterol metabolism is vital for normal liver function.^[^ ^36^ ^]^ However, this metabolism is often impaired in drug‐induced liver injury (DILI). Therefore, studying the intervention effects of DILI and geniposidic acid (GPA) from the perspective of bile acid‐cholesterol metabolism holds significant clinical importance.

The key feature of both APAP‐induced chemical liver injury and TP‐induced acute and chronic herbal liver injury is hepatocellular injury. Previous mechanistic studies using APAP as a classic model of experimental DILI have focused on its inherent toxicity and dose‐dependent therapeutic mechanisms.^[^ ^37^, ^38^, ^39^ ^]^ However, DILI can also occur without dose dependence, with liver injury happening when a certain dose threshold is reached.^[^ ^40^ ^]^ The latency period for this type of DILI can range from days to years, showing a trend toward chronic development. Thus, APAP‐induced DILI is not entirely suitable as an experimental pharmacological model of DILI. In this study, we incorporated TP‐induced acute intermediate DILI as an experimental pharmacological model to simulate DILI that does not show dose‐dependence and has a long latency period, in addition to APAP‐induced chemical DILI. Our study demonstrated that, in addition to hepatocellular injury and the release of inflammatory factors, abnormalities in bile acid and cholesterol metabolism were observed in the TP acute DILI mouse model. Therefore, the therapeutic mechanism of DILI should emphasize bile acid and cholesterol metabolism in the liver, alongside reducing inflammation levels.^[^ ^41^ ^]^ Dynamic monitoring of bile acid and cholesterol changes during the disease course will help in comprehensively understanding and diagnosing the progression of DILI. In DILI, FXR plays a significant regulatory role in bile acid and cholesterol production and metabolism.^[^ ^42^ ^]^ Various FXR agonists, such as GW4064 and OCA, have been used in the treatment of metabolic diseases. However, FXR's ligand‐binding domain (LBD) has a flexible binding cavity, and different ligands produce different conformational changes upon binding.^[^ ^43^ ^]^ These changes can result in the selective recruitment of different co‐binders for downstream target genes, potentially leading to off‐target effects or additional toxicity. Therefore, pharmacodynamic‐based screening of FXR‐specific ligands presents a novel strategy for treating DILI.

In this study, we identified GPA as an FXR‐specific agonist through luciferase reporter assays, demonstrating a significant therapeutic effect in ex vivo models of DILI. By comparing the binding modes of GPA and GW4064 to FXR, we found that GPA's binding to FXR LBD was primarily stabilized by specific hydrogen bonding between Ser332 and His447 residues. Specifically, the hydroxyl group on the cyclic enol ether terpene of GPA formed a hydrogen bond with His447, controlling the distance to Helix‐12 and stabilizing the active conformation capable of recruiting coactivators. This binding pattern was similar to that of GW4064. Molecular docking results showed that the hydroxymethyl group on GPA's sugar ring formed hydrogen bonds with the hydroxymethyl group on the side chains of Met328 and Ser332, while the hydroxyl group on the benzoic acid substituent of GW4064 formed hydrogen bonds with Arg331.^[^ ^44^ ^]^ Both compounds bound to the FXR H11 helix in the hammer region. Notably, GW4064 also interacted with Met265 and Met290, and the differences in binding to these residues may explain differences in drug effects.^[^ ^45^ ^]^ The absence of classical hydrogen bond interaction with Met265 and Met290 in GPA and the different binding residues on helix H11 result in the inward squeezing of helices H5, H3, and H7, shortening the binding region between GPA and FXR LBD and reducing the binding cavity volume.

The hydrogen bond between the hydroxyl group on GPA's iridoid parent core and the N–H moiety in His447 is critical for maintaining FXR's active state, potentially stabilizing the protein's active conformation capable of recruiting coactivators. This step requires experimental verification. In conclusion, GPA binds to FXR LBD through a T‐hammer binding mode, confirming GPA as an FXR ligand at the atomic level and elucidating the conformational changes induced by GPA targeting FXR. AlphaScreen and subsequent experiments revealed that GPA and GW4064 recruit different coactivators. GPA recruited more SRC2‐3, while GW4064 favored PGC1α recruitment. This difference may be related to GPA's binding to Ser332 of FXR LBD, ultimately activating CYPs and miR‐19a‐3p, exerting unique pharmacological effects in DILI treatment.^[^ ^46^ ^]^ We established APAP and TP models of acute and chronic liver injury to explore the therapeutic effect of GPA. GPA altered bile acid‐cholesterol metabolic homeostasis, demonstrating pharmacological activity against DILI. Structural pharmacology studies showed that GPA induces conformational changes in FXR by binding to Ser332 and His447 residues, activating CYPs to promote bile acid metabolism and regulate bile acid synthesis. Additionally, GPA upregulates miR‐19a‐3p expression after FXR activation, reducing bile acid disorder by inhibiting LXR‐mediated cholesterol synthesis.

Here, we demonstrated that GPA targets FXR and show that GPA activates FXR to regulate CYPs‐miR‐19a‐3p. Considering the dual mechanisms of GPA, we believed that transactivation of CYPs or miR‐19a‐3p alone would have limitations. CYPs are key phase I/II metabolic enzymes in the body. Transcriptional activation of CYPs alone may cause unwanted side effects. At the same time, the related therapeutic targeting of miR‐19a‐3p was poor with the available technology. As a ligand‐dependent transcription factor, FXR can be involved in the regulation of bile acid, glucose, and lipid metabolism. Different ligands binding to FXR caused different regulatory trends. Thus, we believed that targeting FXR with GPA to regulate the cascade of “bile acid cholesterol” balance would have a better effect than transcriptional activation of CYP or miR‐19a‐3p.

## 4 Conclusion

Our study provides a new mechanism for diagnosing and treating patients with DILI, suggesting that clinicians should monitor bile acid and cholesterol metabolism in DILI patients and adjust clinical medication accordingly. GPA, a small molecule of Chinese medicinal origin, exerts anti‐DILI effects by targeting FXR, offering high safety and potential for clinical drug development.

## 5 Experimental Section

### Study Design

To investigate whether and how GPA ameliorates DILI, both in vivo and in vitro experiments were carried out in mice with systemic deficiency of FXR, and in hepatocytes after challenge using pharmacological compounds and genetic tools. Liver samples were collected for miRNA sequencing, lipidomics, and bile acid analysis. Molecular docking, thermal transfer, Alphascreen, SPR, and dual luciferase reporter gene assays were performed to assess the binding ability of GPA to FXR.

### Animal Experiment

Male wild‐type (WT) C57BL/6 mice aged 6–8 weeks were purchased from Guangdong Medical Laboratory Animal Center (Guangzhou, China), *Fxr^−/−^* mice were obtained from Jackson Laboratories. FXR silencing was achieved by injecting AAV‐shFXR adenovirus at a dose of 8 × 10^12^ vg kg^−1^ through the tail vein for 20 days. All animals were maintained in a specific pathogen‐free environment, and all animal experiment protocols were approved by the Animal Care Committee of the International Institute of Translational Chinese Medicine, Guangzhou University of Chinese Medicine. The approved number of the Animal Ethics: 20 230 831.

### Acute DILI in Mice

Mice were administered GPA by gavage at low (25 mg kg^−1 ^ d^−1^), medium (50 mg kg^−1 ^ d^−1^), and high doses (100 mg kg^−1 ^ d^−1^), while the vehicle group received an equal volume of saline according to body weight. The drug was administered continuously for 6 days, once daily. To induce drug hepatotoxicity, on day 7, mice were injected intraperitoneally with 400 mg kg^−1 ^ APAP or 1 mg kg^−1 ^ TP following GPA administration, followed by overnight fasting.

### Chronic DILI in Mice

Mice were administered GPA at doses and in a manner as mentioned above. GPA was administered continuously for 21 days, once daily. Chronic liver injury was induced by gavage administration of 0.5 mg kg^−1 ^ TP in all groups except the vehicle group, which received an equal volume of 0.5% CMC‐Na. Blood and liver samples were collected at the indicated time points for further analysis.

### Cell Lines and Stimulations

AML12 (mouse hepatocytes) and L02 (human hepatocytes) cell lines were purchased from Procell (Shanghai, China) and cultured in DMEM/F‐12 and RPMI‐1640 medium respectively supplemented with 10% fetal bovine serum. Both cell lines were stimulated with TP (50 nm) for 24 h. Unless otherwise stated, the following concentrations were used in vitro: GPA (25, 50, and 100 µm), GW4064 (20 µm), and both GPA and GW4064 were administered 12 h after the addition of TP.

### Cell Transfection

AML12 or L02 cells were seeded in 6‐well plates. After 24 h, cells were transfected with a plasmid expressing 3× Flag‐tagged human FXR WT‐pcDNA3.1 (50 nm) using the Lipofectamine 3000 transfection kit. Negative control cells were treated with transfection reagents only, without plasmid. Cells were harvested 48 h after transfection for further experiments. Unless otherwise specified, FXR mutant plasmids, bio‐hsa‐miR‐19a‐3p, hsa‐miR‐19a‐3p mimic/inhibitor, and mmu‐miR‐19a‐3p mimic/inhibitor were used to transfect cells at 50 nm as described above.

### Metabolomics Analysis

Mouse liver samples were collected and immediately stored at −80 °C. Samples were thawed on ice before analysis. ExionLC AD UPLC‐QTRAP instrument was used and analyzed by Metware Biotech.

### Biochemical and Histological Assessment

Liver injury was assessed biochemically and histologically. Serum and liver alanine aminotransferase (ALT), aspartate aminotransferase (AST), alkaline phosphatase (ALP), total bile acids (TBA), total triglycerides (TG), total cholesterol (TCHO), high‐density lipoprotein cholesterol (HDLC), low‐density lipoprotein cholesterol (LDLC), and free cholesterol (FC) activity were determined using commercial kits (Nanjing Jiancheng Bioengineering Institute, China). Hematoxylin and eosin (H&E) staining, oil red O staining, nile red staining, and F4/80 immunohistochemical (IHC) staining were performed on liver sections. Photographs were taken in a blinded fashion at random fields, and representative pictures of liver sections were displayed.

### Fluorescence In Situ Hybridization (FISH) Assay

The FISH assay was performed to observe the location of miR‐19a‐3p. CY5‐labelled probes specific to miR‐19a‐3p were designed and synthesized by Servicebio (Wuhan, China). The sequences of miR‐19a‐3p probes for FISH were as follows: UGUGCAAAUCUAUGCAAAACUGA. Images were acquired using a Leica TCS SP8 fluorescence microscope.

### Luciferase Reporter Assay

For transactivation assays, the pCDNA3.1(+)‐empty vector, pCDNA3.1(+)‐FXR WT, S332A, H447A, and DM S332AH447A (100 ng well^−1^), pGL3‐basic hBSEP‐luc (200 ng well^−1^), and pGL3‐CMVR plasmid (10 ng well^−1^) were transiently transfected into L02 cells in 96‐well plates using Lipofectamine 3000 (Invitrogen, USA). After 24 h of transfection, cells were treated with fresh DMEM (100 µL) containing DMSO, GW4064 (10 µm, #G5172, Sigma), or GPA (25–100 µm) for 24 h. Relative luciferase activities were measured using the Dual‐Luciferase Reporter Assay System (Promega, Madison, WI) according to the manufacturer's protocols.

For miR‐19a‐3p with LXR luciferase reporter assay, cells were co‐transfected with miR‐19a‐3p mimics, inhibitors, and negative controls (50 nm well^−1^), pGL3‐hLXR WT‐3′UTR‐luc (200 ng well^−1^), pGL3‐hLXR MUT‐3′UTR‐luc (200 ng well^−1^), and pGL3‐CMVR plasmid (10 ng well^−1^). Subsequent processing and assays were performed as described above.

### Immunofluorescence Assay

Cells were incubated with FXR primary antibodies, then stained with DAPI in the dark. Images were observed under the Leica TCS SP8 fluorescence microscope.

### Molecular Docking

Molecular docking was performed using Schrödinge's Maestro software version 11.5. The FXR‐LBD protein structure was downloaded from the PDB database (UniProtID Q96RI1) as a receptor grid. Ligands were downloaded from the Pubchem database (geniposidic acid). Ligands were set using LigPrep settings with default settings. Ligand docking and scoring were performed using the default settings of Glide and SP (standard precision) or XP (extra precision) scoring. PyMOL was applied to analyze and visualize docking poses.

### Plasmid Construction

Full‐length human FXR was obtained by RT‐PCR and cloned into pCDNA3.1(+)‐N‐3×Flag vector. Human FXR LBD WT (248‐476) expression plasmid was generated by inserting FXR LBD WT (248‐476) with an N‐terminal 8×His‐Tag into pET28a. The human bile salt export pump (hBSEP) promoter reporter was generated by inserting a genomic DNA fragment upstream of the transcription start site, which contained an FXR responsive element (FXRE), into the pGL3‐basic‐luc vector. The 3′UTR region of the human *lxr* mRNA was generated by inserting a genomic DNA fragment into the pGL3‐basic‐luc vector. Human FXR S332A, H447A, and DM S332AH447A plasmids and pGL3‐hLXR MUT‐3′UTR mutations were constructed using the Quick‐Change assay and appropriate mutant oligos. All plasmids and their mutations were confirmed by nucleic acid sequencing provided by Tsingke.

### Recombinant Protein Expression and Purification

The human FXR LBD (residues 248–486) was expressed with an N‐terminal 8×His‐Tag from the T7 promoter of the pET28a vector. BL21 (DE3) cells transformed with the expression plasmid were grown in LB broth at 37 °C and induced with 0.2 mm Isopropyl β‐D‐thiogalactoside at 16 °C. The cells were harvested and sonicated in buffer A (20 mm Tris, pH 8.0, 300 mm NaCl, 10% glycerol, and 10 mm imidazole) per 4 liters of cells. The lysate was centrifuged at 14 000 rpm for 60 min, and the supernatant was loaded onto a Ni‐NTA beads Gravity Column pre‐equilibrated with 5 times of column volume (CV) of buffer B (20 mm Tris‐HCl pH 8.0, 300 mm NaCl, 10% glycerol, 10 mm imidazole, 5 mm β‐mercaptoethanol, and 1% PMSF). Lysate and beads and were combined for 60 min at 4 °C, then the column was washed sequentially with 2 CV of buffer B. Protein was eluted over a gradient with buffer C (20 mm Tris‐HCl pH 8.0, 300 mm NaCl, 250 mm imidazole, and 1% PMSF). Preliminary purification fractions containing the target protein were pooled and injected onto a HiTrap QHP column equilibrated with QHP buffer A (20 mm Tris‐HCl pH 8.0, 50 mm NaCl, 1 mm DTT). Diluted fractions were then fractionated with a shallow gradient of QHP buffer B (20 mm Tris‐HCl pH 8.0, 1 m NaCl, 1 mm DTT). Finally, pooled fractions with purity >90% protein were concentrated to a final concentration of ≈5 mg mL^−1^. Further purification fractions containing the target protein were pooled and concentrated at 4 °C using Amicon Ultra‐15 centrifugal filter devices with a 30 000 MWCO.

### Thermal Shift Assay

Melting curves of recombinant human FXR LBD protein with and without the presence of GPA were determined by a thermal shift assay using Protein Thermal Shift kits (Thermo Fisher Scientific, Massachusetts, USA), an Applied Biosystems 7500 fast apparatus (Thermo Fisher Scientific, Massachusetts, USA), and clear non‐skirted 96‐well PCR plates. The sigmoidal plot of the normalized fluorescence intensity values versus temperature was fitted to the Boltzmann equation, and the melting temperature (Tm), where 50% of protein is denatured, was determined.

### SPR Assay

Surface plasmon resonance (SPR) analysis was performed on a Biacore T200 system (Biacore, GE Healthcare, Boston, MA, USA) using Sensor S CM5 chips coated with FXR LBD WT, FXR LBD S332A, FXR LBD H447A, or FXR LBD DM S332AH447A protein. The optimum pH for all types of protein enrichment on the chip is 4.5. GPA was dissolved in PBS buffer; the response value of the interaction was detected according to a specific drug concentration gradient. When the response value generated by the combination of the compound and the chip reached saturation, the KD value was determined as the compound concentration corresponding to the half‐saturation response value.

### AlphaScreen Assay

The binding of various coregulator peptide motifs to FXR LBD in response to ligands was determined by AlphaScreen assays from Revvity. The experiments were conducted with ≈20–40 nm receptor recombinant FXR LBD protein and 20 nm biotinylated cofactor peptides in the presence of 5 µg mL^−1^ donor and acceptor beads in 1× Alpha buffer. Biotinylated cofactor peptides included SRC1‐2 (SPSSHSSLTERHKILHRLLQEGSP), SRC2‐3 (QEPVSPKKKENALLRYLLDKDDTKD), SRC3‐3 (PDAASKHKQLSELLRGGSG), PGC1α (AEEPSLLKKLLLQP), and NCoR‐2 (GHSFADPASNLGLEDIIRKALMGSF). EC_50_ values for the effects of ligand binding to FXR LBD were constructed from a nonlinear least square fit of the data based on an average of three repeated experiments.

### miRNA Sequencing (RNA‐Seq) Analysis

The transcriptome sequencing and analysis were conducted by Metware Biotech. The differential expression of miRNA was analyzed using EdgeR software, with the dispersion set to 0.01. The screening criteria for differential miRNA were a change in the expression level of more than twofold compared to the control and *P*‐value < 0.05.

### Nuclear and Cytoplasmic RNA Extraction

Nuclear and cytoplasmic fractions were isolated using a Beyotime kit and RNase inhibitor. Briefly, L02 and AML‐12 cells were lysed in a Cell Fraction Buffer on ice for 10 min. After centrifugation at 500 g for 3 min at 4 °C, the supernatant was collected as the cytoplasmic fraction. The pelleted nuclei were washed with a Cell Fraction Buffer and used as the nuclear fraction. The cytoplasmic and nuclear components were separately processed with Trizol for total RNA extraction.

### miRNA Pulldown Assay

The miR‐19a‐3p probe and negative control (NC) probe were biotinylated, and the expression of LXR bound to the pulldown beads was measured by RT‐qPCR. Cell extracts (2 µg) were mixed with biotinylated RNA (100 pmol). Washed streptavidin agarose beads (100 µL) were added to each binding reaction and incubated at room temperature for 1 h. Total RNAs and controls were also assayed to confirm that the detected signals were from RNAs specifically binding to miR‐19a‐3p.

### Western Blot Analysis

Extracted proteins were quantified as previously described. Proteins were assessed with the following primary antibodies (Abs). Abs to FXR (48 kDa, 25055‐1‐AP), LXR (48 kDa, 14351‐1‐AP), CYP7B1 (55 kDa, 24889‐1‐AP), CYP27A1 (58 kDa, 67045‐1‐Ig), BSEP(110 kDa, 14351‐1‐AP) were purchased from Proteintech. Abs to CYP7A1 (37 kDa, #DF2612), CYP8B1 (37 kDa, #DF4762) were purchased from Affinity Biosciences. The protein levels were normalized using the anti‐GAPDH antibody (37 kDa, GB11002) purchased from Servicebio.

### Quantitative Real‐Time PCR (qPCR) Analysis

Total RNA was extracted by Trizol (Invitrogen). The reverse transcription and qPCR reactions were conducted with Prime‐Script RT and SYBR RT‐PCR kits (Takara, Japan) following the manufacturer's instructions. The amount of mRNA was calculated based on the *ΔΔC_T_* method and normalized by *gapdh*. The expression of genes involved in bile acid synthesis and transport (*cyp7a1, cyp7b1, cyp8b1, cyp27a1, bsep, mrp2, ntcp*), inflammation cytokine (*tnfα, il‐1β, il‐6, il‐10*), cholesterol synthsis, was and transport (*lxr, srebp1c, fas, scd, apob, abca1, abcg5, ldlr*) and others (*fxr, mdr2, tgr5, glp‐1, pxr, s1rp2, erk, vdr, mek*).

### Statistical Analysis

Data were analyzed via GraphPad Prism 9.0 software and were presented as means ± SD. Independent sample *t‐*test or one way analysis of variance (ANOVA) were as necessary, with *P* < 0.05 being statistically significant.

### Ethics Approval and Consent to Participate

All instructional and National Guidelines for the care and use of animals (fisheries) were followed.

## Conflict of Interest

The authors declare no conflict of interest.

## Author Contributions

M.F. dealt with the conceptualization, investigation, writing the original draft, and data curation. Y.X. dealt with the software, validation, and formal analysis. B.W. dealt with the investigation and formal analysis. J.L. dealt with the data curation. C.L. dealt with the methodology, writing review, and editing. Z.L. dealt with the visualization. R.Z. dealt with the supervision and funding. Z.L. dealt with the supervision and funding. C.W. dealt with conceptualization, methodology resources, writing the original draft, supervision, and funding.

## Data Availability

Research data are not shared.
